# Behavioral and Neural Plasticity of Ocular Motor Control: Changes in Performance and fMRI Activity Following Antisaccade Training

**DOI:** 10.3389/fnhum.2015.00653

**Published:** 2015-12-18

**Authors:** Sharna D. Jamadar, Beth P. Johnson, Meaghan Clough, Gary F. Egan, Joanne Fielding

**Affiliations:** ^1^Australian Research Council Centre of Excellence for Integrative Brain Function and Monash Biomedical Imaging, Monash UniversityMelbourne, VIC, Australia; ^2^School of Psychological Sciences and Monash Institute of Cognitive and Clinical Neurosciences, Monash UniversityMelbourne, VIC, Australia; ^3^Department of Medicine, University of MelbourneMelbourne, VIC, Australia

**Keywords:** antisaccade, fMRI, training, ocular motor, motor control

## Abstract

The antisaccade task provides a model paradigm that sets the inhibition of a reflexively driven behavior against the volitional control of a goal-directed behavior. The stability and adaptability of antisaccade performance was investigated in 23 neurologically healthy individuals. Behavior and brain function were measured using functional magnetic resonance imaging (fMRI) prior to and immediately following 2 weeks of daily antisaccade training. Participants performed antisaccade trials faster with no change in directional error rate following 2 weeks of training; however this increased speed came at the cost of the spatial accuracy of the saccade (gain) which became more hypometric following training. Training on the antisaccade task resulted in increases in fMRI activity in the fronto-basal ganglia-parietal-cerebellar ocular motor network. Following training, antisaccade latency was positively associated with fMRI activity in the frontal and supplementary eye fields, anterior cingulate and intraparietal sulcus; antisaccade gain was negatively associated with fMRI activity in supplementary eye fields, anterior cingulate, intraparietal sulcus, and cerebellar vermis. In sum, the results suggest that following training, larger antisaccade latency is associated with larger activity in fronto-parietal-cerebellar ocular motor regions, and smaller antisaccade gain is associated with larger activity in fronto-parietal ocular motor regions.

## Introduction

Saccadic eye movements have been used extensively as an experimental tool to gain insight into the processes that govern motor control; in particular, the cognitive processes underlying goal directed behavior. Of the many paradigms devised to investigate these processes, one of the most widely used is the antisaccade task, which evaluates the capacity to inhibit a reflexive response in favor of a complex volitional behavior. This task is a simple variation of the visually-guided, or prosaccade task: where a prosaccade involves a shift of gaze to a newly presented stimulus; a correctly executed antisaccade requires an individual to refrain from looking at a sudden-onset peripheral target, and instead direct gaze to its mirror image location.

The antisaccade task is under investigation as a potential endophenotype or biomarker for a number of psychiatric and neurodegenerative conditions. While neurologically healthy individuals often fail to inhibit a reflexive response to the target stimulus on a substantial number of trials (e.g., Schaeffer et al., 2013), referred to as antisaccade directional errors, a significant body of work has shown that antisaccade directional error rates increase significantly for psychiatric and neurodegenerative patient populations (see Hutton and Ettinger, 2006 for a review). Further, antisaccade latencies are often prolonged or more variable, and spatial accuracy compromised in these individuals. Consequently, performance on the antisaccade task has been proposed as potentially useful measure of disease severity, progression, and therapeutic effect of treatment for many of these disorders.

**Figure 1:**
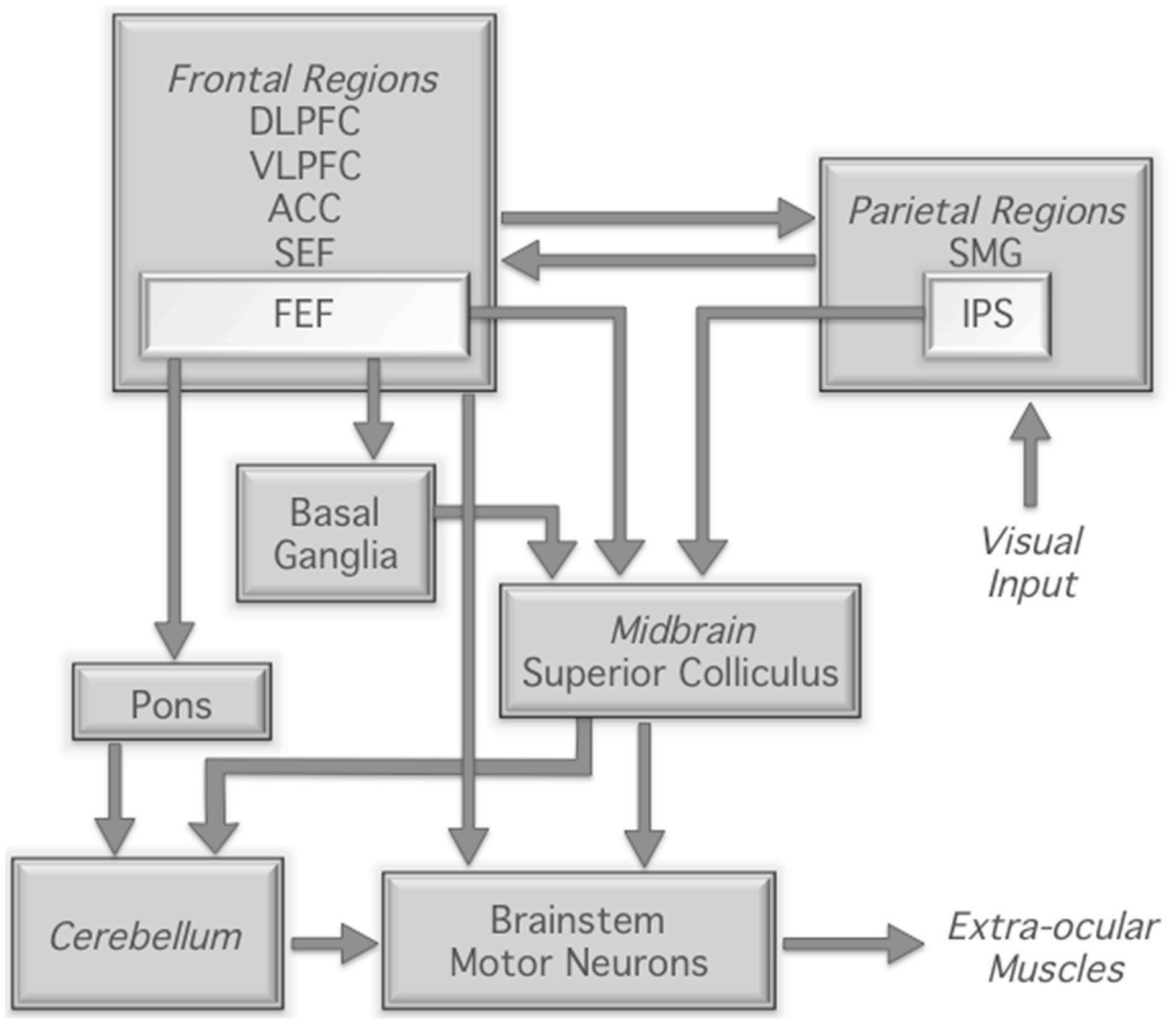
**Neural regions involved in the control of saccadic eye movement; based on Munoz and Everling (2004)**. DLPFC, dorsolateral prefrontal cortex; VLPFC, ventrolateral prefrontal cortex; ACC, anterior cingulate cortex; SEF, supplementary eye fields; FEF, frontal eye fields; SMG, supramarginal gyrus; IPS, intraparietal sulcus.

The neural correlates of the antisaccade task have been widely investigated using functional magnetic resonance imaging (fMRI). These studies have demonstrated robust and reliable activation of a broadly distributed network of regions implicated in the generation and control of eye movements more generally (Figure 1). Specifically, compared to prosaccade or fixation trials, antisaccade trials consistently show increased fMRI activity in the intraparietal sulcus, supplementary and frontal eye fields, dorsolateral prefrontal cortex, anterior cingulate cortex, thalamus, striatum, and cerebellum (Curtis and D'Esposito, 2003; DeSouza et al., 2003; Ford et al., 2005; McDowell et al., 2005; Dyckman et al., 2007; Ettinger et al., 2008; Hwang et al., 2010). The most consistent finding in the human functional neuroimaging literature is increased frontal and supplementary eye field activation for antisaccades compared to prosaccades (Jamadar et al., 2013). The frontal and supplementary eye fields are involved in preparing the voluntary antisaccade response (Pierrot-Deseilligny et al., 2004) and may be involved in biasing the oculomotor system for an antisaccade response over the prepotent prosaccade response (e.g., Schlag-Rey et al., 1997). The thalamus and striatum are critical subcortical components of the cortico-subcortical motor networks (e.g., Parent and Hazrati, 1995), interacting with cortical eye fields to form the cortico-thalamic-striatal oculomotor networks involved in reflexive and voluntary eye movements (Isoda and Hikosaka, 2011). The dorsolateral prefrontal cortex is involved in the top-down biasing of the oculomotor system in the service of task goals (Pierrot-Deseilligny et al., 2003, 2004; Ford et al., 2005; Brown et al., 2006; Ettinger et al., 2008; Hwang et al., 2010) and the anterior cingulate is involved in signaling the requirement for increased cognitive control for the more conflict- and interference-prone antisaccade trials (e.g., Botvinick et al., 2004). Finally, the cerebellum, particularly the cerebellar vermis, is crucially involved in the fine motor control of saccadic eye movements (Robinson and Fuchs, 2001).

The stability of antisaccade performance in neurologically healthy individuals, crucial to its use as a biomarker or endophenotype in patient populations, has been investigated in only a few studies. While some studies have demonstrated within-subject stability of antisaccade latencies over periods of 1–2 months (Roy-Byrne et al., 1995; Klein and Berg, 2001; Ettinger et al., 2003; Blekher et al., 2009), reports of within-subject stability of antisaccade directional errors in test-retest paradigms are modest. For example, Ettinger et al. (2003) revealed a significant decrease in antisaccade errors across two testing sessions conducted 2 months apart (from 20.9 to 16.4%). The authors attributed these differences to practice effects, with the first session serving as practice for the second session.

To date, studies that have examined training effects on the antisaccade task have used training paradigms that required a peripheral motor response (i.e., button press) instead of a saccadic response. Dyckman and McDowell (2005) trained participants over 2 weeks on antisaccade, prosaccade, or fixation tasks using a button press version of the tasks (Dyckman and McDowell, 2005). Specifically, participants were instructed to move their attention and their eyes to the opposite side of a screen to identify with a button press a briefly presented target. Participants who trained on the button-press antisaccade task significantly decreased their antisaccade error rates with no change in antisaccade latencies. However, they also found that training using the fixation protocol reduced antisaccade latency, while training on the button-press prosaccade task led to increased antisaccade errors. Thus, in individuals trained on the button-press antisaccade task, training on a related but not identical task resulted in improved antisaccade performance.

Using a similar button press antisaccade task, Unsworth et al. (2011) found reduced error rates (32 to 8%) and shorter latencies in 25 healthy adults following extensive training (14 blocks of 250 trials). The authors proposed that following training, improvement was seen for both the inhibition of a prosaccade and the generation of an antisaccade. Improvement was also seen for goal maintenance processes (working memory processes), with practice allowing for automaticity to build for each of these processes/behaviors in parallel.

To date, only one study has investigated the underlying neural changes that accompany the behavioral effect of antisaccade training. Using the button-press training design of Dyckman and McDowell (2005), Lee et al. (2012) found significant reductions in error rate and latency on the button-press but not saccade version of the antisaccade task. However, for the button-press antisaccade training group, training resulted in a reduced number of activated voxels in the frontal and supplementary eye fields, the superior parietal lobule, and cuneus. As these fMRI changes were obtained in the absence of behavioral changes, the authors concede that these changes may reflect improved cognitive control more generally, rather than an improvement in antisaccade performance *per se*.

The inconsistent previous results may be attributable to these studies using a different paradigm for the training vs. test sessions, with the training paradigms requiring a button press response, and test paradigms requiring an ocular motor response only. Previous studies of transfer of cognitive training improvements suggest that acquired skills are often specific to the practiced task, or to tasks that rely on almost identical processes and networks (Dahlin et al., 2008; Jolles et al., 2010). Furthermore, each of these studies used repetitive training paradigms, where each group of subjects was trained on a single task only (e.g., antisaccade, prosaccade, or fixation trials). It is known that practicing tasks with increased contextual interference, for example by introducing trials that interfere with the trial type of interest, and interleaving trial types in a randomized order, results in superior retention and transfer of learning effects compared to repetitive task practice (Shea and Morgan, 1979; Schmidt and Bjork, 1992). Improved learning effects with interleaved trial presentation are associated with enhanced functional connectivity within task-relevant regions compared to repetitive trial presentation (Lin et al., 2013).

In sum, it remains to be established whether training on an ocular motor antisaccade saccade task results in behavioral and neural changes in the ocular motor network over time. Our study adopted an antisaccade training protocol that was optimized to induce learning effects. Our training paradigm replicated the saccadic paradigm used in the imaging sessions with different trial order to control for sequence learning. Antisaccade and prosaccade trials were interleaved to investigate functional changes associated with training. We hypothesized that training would be associated with changes in activity in the ocular motor network (Figure 1), and that these changes would be associated with changes in antisaccade performance measures. Given that training on cognitive paradigms usually results in reduced activity in the task-related network (Kelly and Garavan, 2005), we hypothesized that activity in the ocular motor network would decrease following training.

## Methods

This work was undertaken with the understanding and written consent of each participant, with the approval of the Monash University Human Research Ethics Committee, and in accordance with the Code of Ethics of the World Medical Association (Declaration of Helsinki) for experiments involving humans.

### Participants

Twenty-three healthy individuals (aged 18–43 [average 25.8] years, 11 male) participated in this study. All participants had normal or corrected-to-normal vision and had no current or previous neurological or psychological illness or injury; women were excluded for current or suspected pregnancy. Participants had no prior exposure to the antisaccade or prosaccade tasks prior to participation.

### Stimuli and tasks

**Figure 2:**
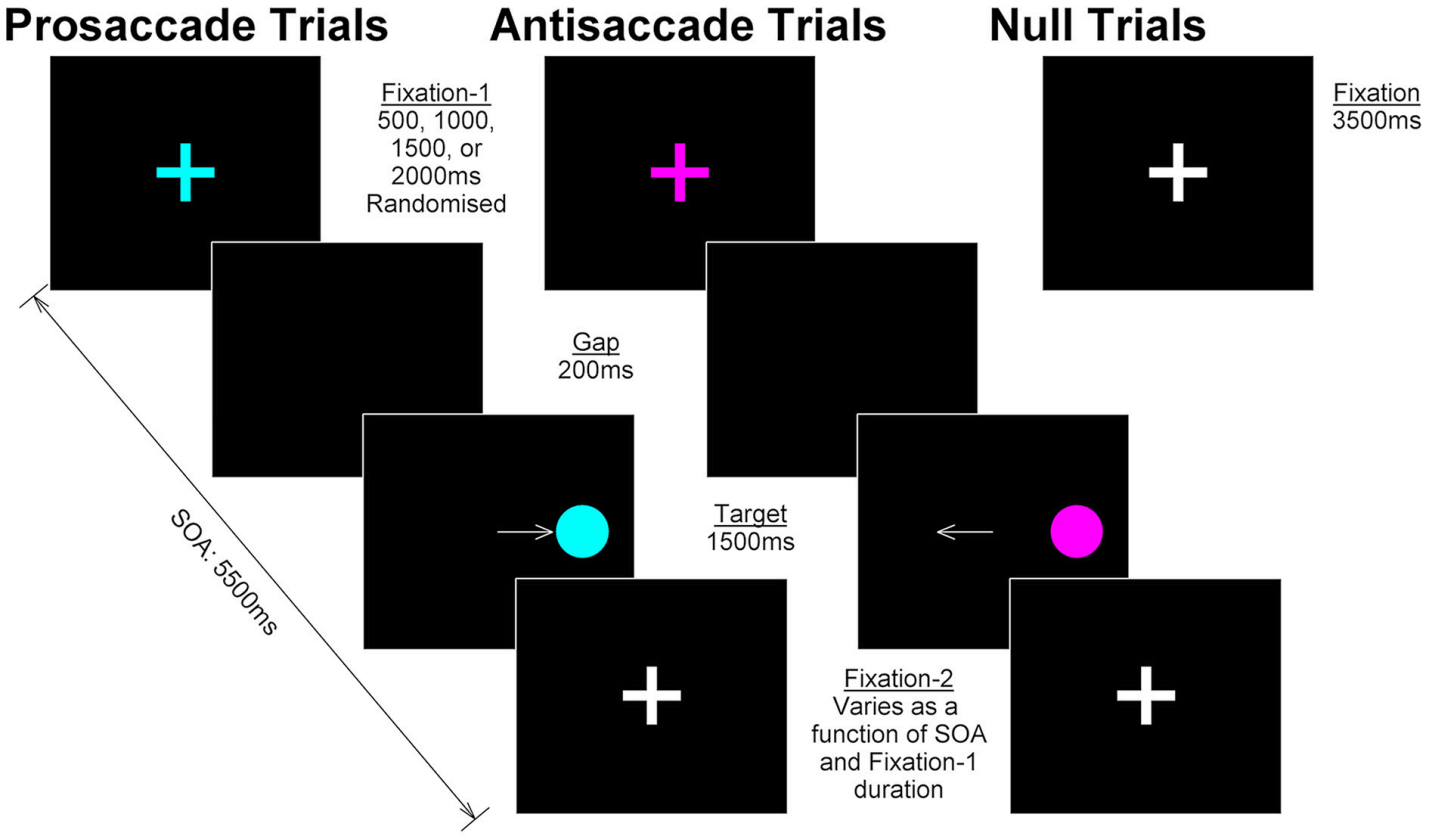
**Trial design**. In prosaccade and antisaccade trials, a colored fixation cross (“fixation-1”) was presented for 500, 1000, 1500, or 2000 ms, randomized between trials; color cued the identity of the currently relevant task and was counterbalanced across participants. Following a 200 ms gap, the target appeared in either the left or right hemisphere (duration 1500 ms); participants were required to make a prosaccade or antisaccade and hold their gaze in that position until the target was removed. The target was replaced by a white fixation cross (“fixation-2”) for the remainder of the SOA (5500 ms—fixation-1 duration—target duration). Null trials consisted of a white fixation cross (duration 3500 ms) visually indistinguishable from fixation-2 in prosaccade and antisaccade trials.

The task was programmed in Experiment Builder v.10 (SR Research, Ontario Canada). Antisaccade, prosaccade, and null trials were presented in pseudorandomised order (no more than four repetitions of the same task, no runs of consecutive nulls, even number of right and left targets within task, no more than four consecutive targets in the same hemisphere) across four blocks in an event-related fMRI design. Participants completed 96 trials of antisaccade, 96 trials of prosaccade and 28 (15%) null trials in each block (total 384 antisaccade, 384 prosaccade, and 112 null trials). Figure 2 shows the trial design. The duration of antisaccade and prosaccade trials was fixed at 5500 ms. Each trial began with the presentation of a fixation cross (subtending 1.75° visual angle; “fixation-1”) on a black background presented for 500, 1000, 1500, or 2000 ms randomized between trials. Fixation-1 was removed and followed by a blank screen (200 ms), after which the target (filled circle subtending 1.75° visual angle with a 0.5° cross hair in center) appeared either 7° from center in either hemifield for 1500 ms. The target was followed by a fixation cross (subtending 1.75° visual angle; “fixation-2”) until the end of the trial (duration varied as a function of fixation-1 duration). For antisaccade and prosaccade trials, fixation-1 and the target were colored in one of two cue colors (e.g., magenta = antisaccade, turquoise = prosaccade or vice versa, counterbalanced between individuals); fixation-2 was always white. Null trials consisted of a white fixation cross, presented 3500 ms and visually indistinguishable from fixation-2; thus participants could not identify when a null trial was in progress. Participants were instructed to fixate on the central fixation until they were sure which way to look; to fixate on the target for the duration of target presentation for prosaccade trials; and to look in the mirror opposite location for the duration of target presentation antisaccade trials.

### Procedure

Participants completed two testing and 14 training sessions. Testing session one consisted of neuropsychological testing followed by MRI scanning with the following protocol: task functional scan (24 min), diffusion tensor imaging (DTI, 21 min), resting state functional scan (5 min) and structural scan (7 min). DTI and resting state results will be reported in a forthcoming manuscript. Testing session one was followed by 14 training sessions, which consisted of shortened versions of the event-related fMRI task described above (15 min, approximately 50 trials of antisaccade and 50 trials of prosaccade). Participants completed training once per day at a time and location of their choice and training was not monitored. All participants except one reported completing the training on every day, the remaining participant completed nine sessions. This subject's performance data was not identified as an outlier, and so was included in the analysis. Testing session two was identical to testing session one. Participants never completed the same sequence of antisaccade, prosaccade, and null trials during testing or training sessions to avoid implicit sequence learning effects.

### Data acquisition and analysis

#### Ocular motor data

Horizontal displacement of the eye was recorded simultaneously with fMRI using an MR-compatible video-based SR Research EyeLink 1000 system, with a spatial resolution of 0.01° and a sampling rate of 500 Hz.

The ocular motor data was analyzed using in-house software to mark the time and location of target onset and offset, as well as saccade onset and offset. The onset of the saccade was defined as the time when eye velocity exceeded 30°/s; the end of a saccade was defined as the time after saccade onset when eye velocity fell below 10°/s. Each trial was manually inspected to ensure correct placement of target and saccade markers, as well as to screen for any errors. Trials were excluded from further analysis if they exhibited blinks prior to 100 ms of the target onset or during the primary saccade, unstable or poor fixation on the centrally presented target (±0.5° from center), small saccades with amplitude < 3° or anticipatory eye movements (saccades made within 100 ms of the peripheral target appearing).

Latency of the primary saccade was defined as the time difference between target onset and the primary saccade onset. Directional errors were defined as trials in which a prosaccade was made during an antisaccade trial, or in which an antisaccade was made during a prosaccade trial. The proportion of trials in which a directional error was made was calculated for both pro- and antisaccades as the [number of trials with a directional error/total number of trials analyzed]^*^100. Primary saccade gain was defined as [saccade amplitude/target amplitude].

Session effects of latency, directional error rate and gain were analyzed for antisaccade and prosaccade trials separately using paired *t*-tests. Ocular motor data was systematically screened for outliers, and subject data identified as ±3 standard deviations above/below the mean were classified as outliers and removed from that *t*-test. Two outliers were identified: one for antisaccade directional error for session 1, and one for prosaccade latency for session 2.

#### MR image data

Magnetic resonance images were acquired on a Siemens Skyra 3 T scanner using a 20 channel head coil. Functional MRI was acquired using a T2^*^-weighted GRAPPA echo-planar imaging (EPI) sequence (ascending axial acquisition, 116 volumes, *TR* = 2.5 s, *TE* = 30 ms, FOV = 192 mm, acquisition matrix = 64 × 64, 44 slices, 3 × 3 × 3 mm voxels). Structural MRI was acquired using a T1-weighted 3D MPRAGE sequence (*TR* = 1900 ms, *TE* = 2.43 ms, flip angle = 9°, FOV = 192 × 192 mm, voxel size = 0.6 × 0.6 × 0.6 mm, 256 slices).

MRI data was analyzed with SPM8 (Wellcome Department of Cognitive Neurology, London). Data for each testing session were preprocessed separately. For functional runs, the first five images were discarded to account for T1 saturation effects. EPI slice acquisition timing differences were corrected using the central slice as reference, realigned to the first image and co-registered to each individual's structural scan. Structural scans were then segmented using the unified segmentation algorithm in SPM8 to derive parameters to normalize from individual subject to MNI space. Functional and structural scans were then normalized to the MNI template using these parameters and spatially smoothed using a 6 × 6 × 6 mm^3^ FWHM Gaussian kernel. For all participants, motion was less than a voxel and the quality of registration was checked.

Antisaccade and prosaccade events for each participant were categorized as correct, corrected directional errors, and uncorrected directional errors. Trials where it was unclear if a response was correct or incorrect on the basis of ocular motor recording (small saccades, blinks, unstable baseline, signal loss, no response) or where participants made anticipatory saccades were collapsed into a nuisance variable. On average, participants generated 73 antisaccade, 79 prosaccade, 10 corrected directional errors (antisaccade), 6 corrected directional errors (prosaccade), 4 uncorrected directional errors (antisaccade), 4 uncorrected directional errors (prosaccade), and 16 nuisance trials per testing session. Due to the small trial numbers for corrected and uncorrected directional errors, these were not further analyzed.

First-level analyses consisted of a model with the seven experimental regressors and six realignment parameters convolved with a haemodynamic response with temporal and dispersion derivatives. Images for each session were entered into the first-level model as separate sessions. At the second-level, contrast images for antisaccade > baseline averaged over session and antisaccade > baseline for each session separately were entered into two second-level random effects analyses. Effects of task (antisaccade) were tested with a one-sample *t*-test and effects of session were tested using a paired samples *t*-test. Results are thresholded at voxel-wise false discovery rate (FDR) corrected *p* < 0.01, with an uncorrected cluster extent threshold *p* < 0.05 to remove very small clusters.

Regions of interest (ROIs) were defined a priori on the basis of the ocular motor network described in Figure 1 in a two-stage process. Firstly, anatomical masks of the region were created using the WFU_PickAtlas (v3.0.4, Maldjian et al., 2003); all regions except the intraparietal sulcus, frontal and supplementary eye fields were anatomically defined according to the AAL atlas. Intraparietal sulcus, frontal and supplementary eye fields were defined using masks obtained from the meta-analysis reported by Jamadar et al. (2013). For the cerebellum, masks were created for the vermis, given its known role in the antisaccade task (Robinson and Fuchs, 2001). Masks were not created for brainstem motor neurons or the superior colliculus due to the difficulty in imaging these deep structures without artifact (Petit and Beauchamp, 2003). In sum, the following masks were created for left and right hemispheres separately: dorsolateral prefrontal cortex, ventrolateral prefrontal cortex, anterior cingulate cortex, frontal eye fields, supplementary eye fields (medial, not bilateral), supramarginal gyrus, intraparietal sulcus, basal ganglia (caudate, putamen), pons, vermis (medial, not bilateral). Secondly, these anatomical masks were used to inclusively mask the antisaccade greater than baseline comparison (averaged over session) and thresholded at FDR corrected *p* < 0.01. The coordinate of maximum activity within the mask was then selected and a spherical ROI (10 mm) created around this peak of activity. **Table 3** gives MNI coordinates for each ROI.

MarsBaR (v0.43, Brett et al., 2002) was used to extract parameter estimates (contrast values) for the antisaccade greater than baseline comparison from each of the ROIs for each individual and session separately. Effects of session and correlations with behavioral measures were tested using SPSS v22 (IBM Corp. New York). The effect of session was tested using paired-samples *t*-tests. Correlations were conducted between each ROI and antisaccade latency, directional error rate and gain for each session separately. Change in correlation strength between sessions was tested using confidence intervals (Zou, 2007). Paired *t*-tests and correlations were corrected for the number of ROIs using FDR adjusted *q* (Benjamini and Hochberg, 1995). Region labels in Tables were determined using the AAL atlas in xjview (http://www.alivelearn.net/xjview8/).

As the main focus of this study was the effects of training on antisaccade performance, results for prosaccade performance are given in Supplementary Materials.

## Results

### Ocular motor results

**Figure 3:**
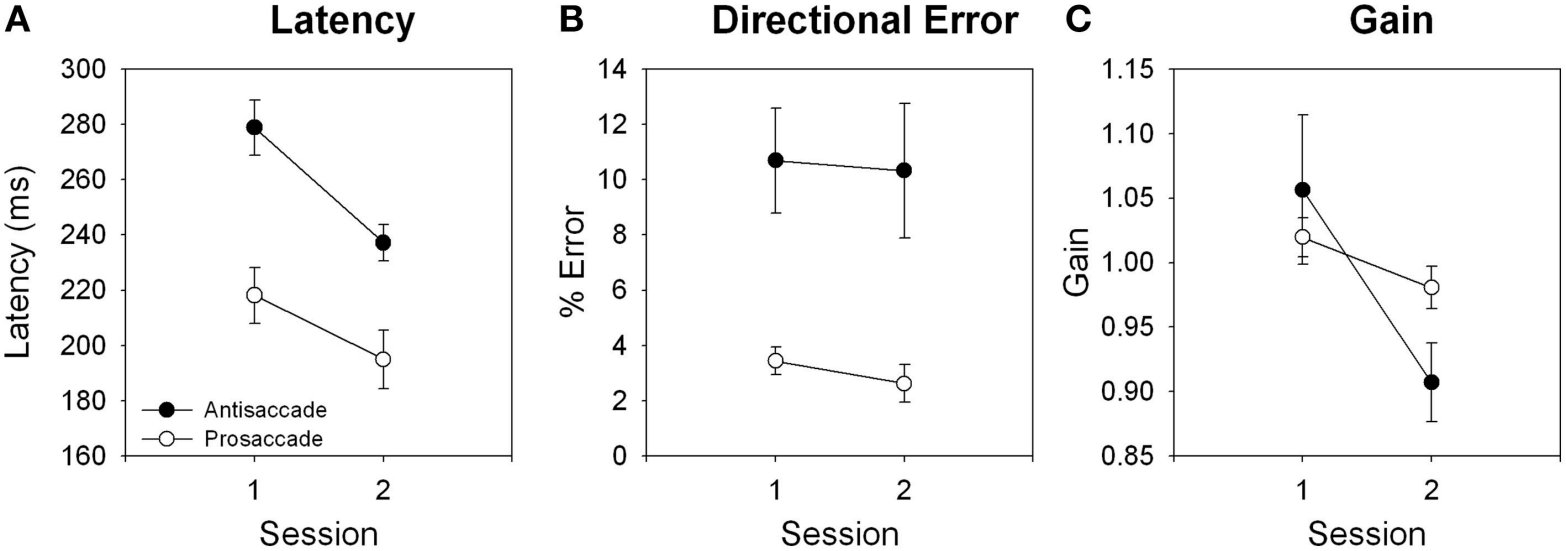
**Effects of training for (A) latency, (B) directional error, and (C) gain for antisaccade and prosaccade trials**.

Ocular motor results are shown in Figure 3. Latency reduced from session 1 to session 2 for antisaccade, *t*_(22)_ = 4.68, *p* < 0.001; and prosaccade, *t*_(21)_ = 4.40, *p* < 0.001, trials. Gain reduced from session 1 to session 2 for antisaccade, *t*_(22)_ = 3.64, *p* = 0.001, and prosaccade, *t*_(22)_ = 2.88, *p* = 0.009, trials. Directional error rate did not differ between session 1 and 2 for either trial type, both *p* > 0.128.

### fMRI results

**Figure 4:**
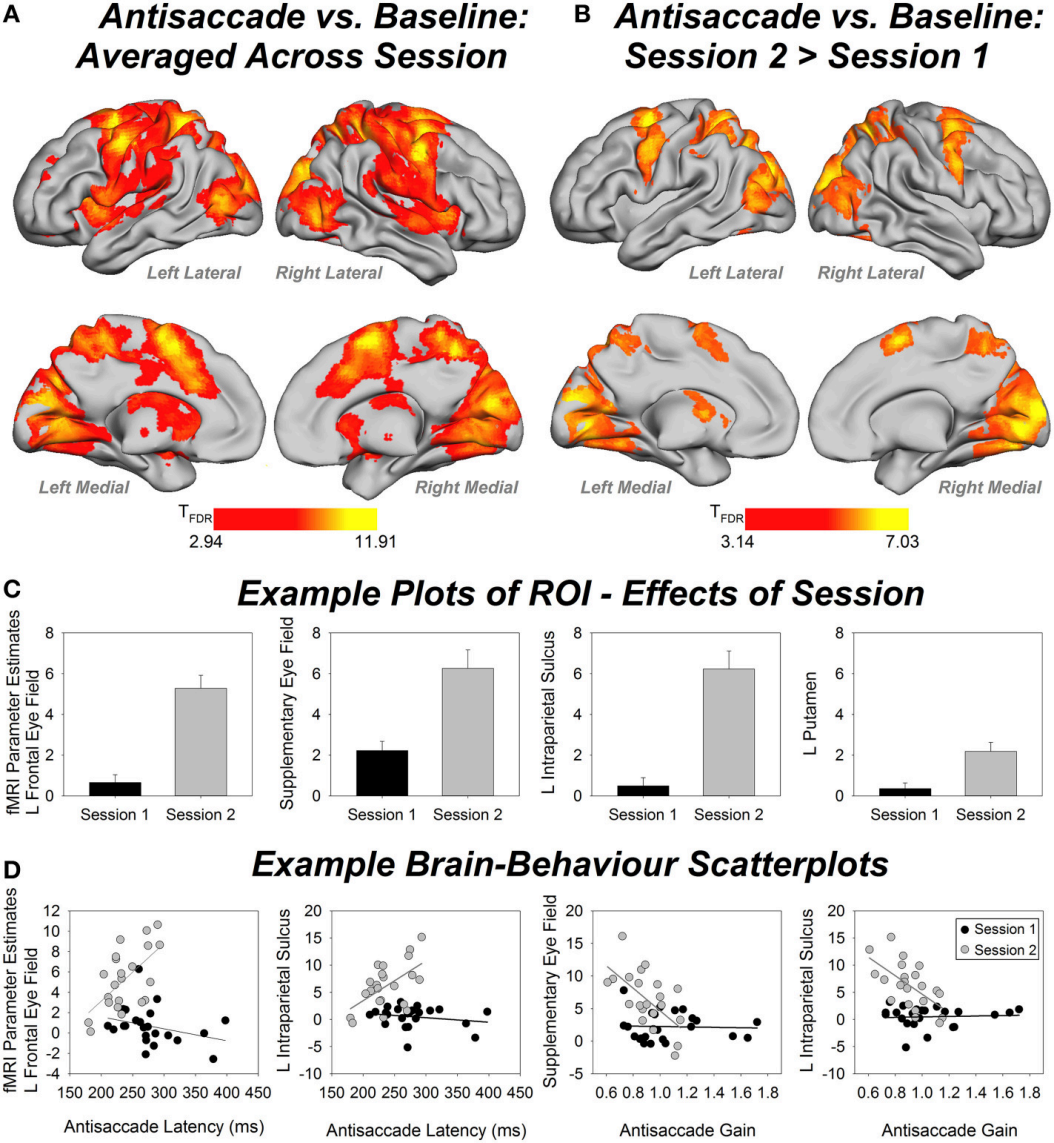
**(A)** Whole-brain fMRI activity for the antisaccade vs. baseline condition, averaged over session. Contrast thresholded at FDR corrected *p* < 0.01, extent threshold *p* < 0.05. **(B)** Whole-brain fMRI activity for antisaccade session 2 > session 1. Contrast thresholded at FDR corrected *p* < 0.01, extent threshold *p* < 0.05. **(C)** Example bar plots for effects of session (fMRI parameter estimates) in four regions of interest (ROIs). Error bars show standard error. **(D)** Example scatterplots for brain-behavior correlations for session 1 (black) and session 2 (white). Y-axis shows fMRI parameter estimates. L, left.

Figure 4A shows whole-brain fMRI results and Table 1 gives region labels, peak MNI and *t*-values for the antisaccade compared to baseline comparison, averaged over session. Antisaccade trials activated a bilateral fronto-basal ganglia-parietal-cerebellar network consistent with previous studies (Jamadar et al., 2013), and with our hypothesized regions of activity. Figure 4B shows fMRI results and Table 2 gives region labels, peak MNI and *t*-values for the effect of session for antisaccade compared to baseline comparison. Antisaccade trials showed increased activity in the fronto-basal ganglia-parietal cerebellar network in session 2 relative to session 1. No region showed larger activity in session 1 vs. session 2^1^.

**Table 1:** **Peak MNI coordinates, ***t***-values, and region labels for the antisaccade > baseline whole brain contrast, averaged over session**.

**Cluster #**	**# Voxels**	**Region label (BA)**	**Peak MNI**	***T*-value**
1	22586	L cuneus (31/18/19)	−27, −70, 16	11.91
		R frontal eye field/precentral gyrus (6)	27, −13, 52	10.50
		L frontal eye field/precentral gyrus (6)	−27, −4, 58	8.32
		L middle occipital gyrus (18/19)	30, −82, 28	7.70
		L IPS/superior parietal lobule (7)	−24, −55, 55	7.97
		R lingual gyrus (18)	18, −76, −11	5.96
		L supplementary eye field	−6, 2, 58	7.70
		R supplementary eye field	3, −1, 61	8.80
		L lingual gyrus (18)	−18, −76, −11	7.63
		L precuneus (7)	−18, −52, 61	8.96
		R IPS/superior parietal lobule (7)	27, −55, 58	6.86
		R cuneus (31/18/19)	18, −85, 25	8.61
		L cerebellar declive (lobule 6)	−24, −64, −26	5.37
		L superior occipital gyrus (18/19)	−21, −79, 28	9.26
		R middle occipital gyrus (18/19)	−18, −82, 19	7.39
		R supramarginal gyrus (40)	27, −40, 46	6.71
		R superior occipital gyrus (18/19)	21, −82, 37	8.86
		R cerebellar declive (lobule 6)	24, −64, −26	5.70
		R precuneus (7)	18, −49, 52	10.03
		R superior temporal gyrus (22)	63, −7, −5	5.25
		L superior temporal gyrus (22)	−54, 2, −2	6.71
		R putamen	36, 11, 4	5.62
		L insula	−36, 10, 3	6.77
		R anterior cingulate (24/32)	6, 17, 40	6.62
		L putamen	−24, −4, 10	6.08
		L cerebellar tonsil (lobule 8)	−33, −52, −41	5.64
		L DLPFC/middle frontal gyrus (9/46)	−12, 23, 34	5.86
		L supramarginal gyrus (40)	−42, −34, 31	6.83
		R insula	39, 11, 4	6.66
		L anterior cingulate (24/32)	−9, 5, 52	7.91
		R cerebellar tuber/declive/uvula (Crus 1)	45, −58, −38	5.59
		R cerebellar tonsil (lobule 8)	33, −52, −41	4.74
		R middle temporal gyrus (39)	−48, −73, 13	6.24
		R fusiform (19)	24, −70, −11	5.72
		L cerebellar tuber/declive/uvula (Crus 1)	−42, −58, −38	5.59
		L caudate	−21, −19, 22	5.18
		R DLPFC/middle frontal gyrus (9/46)	54, −1, 24	6.45
		L cerebellar culmen (lobule 4/5)	−33, −52, −38	6.17
		L thalamus	−15, −22, 16	5.65
		L fusiform gyrus (19)	−24, −76, −11	5.65
		R caudate	18, 14, 1	5.31
		cerebellar vermis (lobule 4/5/6/8)	6, −70, −11	8.87
		L inferior frontal gyrus (44/47)	−60, 5, 13	5.59
		R thalamus	18, −16, 10	5.38
		R VLPFC/inferior frontal gyrus (44/47)	−36, 11, 7	5.93
		L middle temporal gyrus (39)	42, −73, −2	6.60
		R cerebellar culmen (lobule 4/5)	36, −55, −35	5.74

*Contrast was thresholded at FDR corrected p < 0.01, extent threshold p < 0.05 (65 voxels)*.

**Table 2:** **Peak MNI coordinates, ***t***-values and region labels for the antisaccade session 2 > session 1 whole brain contrast**.

**Cluster #**	**# Voxels**	**Region Label (BA)**	**Peak MNI**	***T*-value**
1	10500	L frontal eye field/precentral gyrus (6)	−27, −1, 67	7.03
		L middle occipital gyrus (18/19)	−24, −85, 25	6.21
		L IPS/superior parietal lobule (7)	−24, −58, 55	6.11
		R lingual gyrus (19)	18, −76, −14	6.26
		R middle occipital gyrus (18/19)	30, −79, 31	5.70
		R IPS/superior parietal lobule (7)	27, −55, 58	6.20
		L lingual gyrus (19)	−18, −76, −14	6.42
		R frontal eye field/precentral gyrus (6)	30, −4, 49	6.08
		L cerebellar declive/culmen (lobule 6)	−24, −64, −29	4.53
		R superior occipital gyrus (19)	15, −97, 16	6.89
		R cuneus (19)	12 −97 16	6.80
		R cerebellar declive (lobule 6)	33, −58, −23	4.65
		L superior occipital gyrus (19)	−24, −85, 28	6.45
		L cuneus (18/19/31)	−15, −85, 16	5.54
		R fusiform gyrus (19)	21, −76, −14	6.64
		R supplementary eye field	3, −1, 61	4.35
		L precuneus (7)	−15, −61, 61	5.48
		L cerebellar culmen/tuber (crus 1)	−33, −55, −32	4.42
		R precuneus (7)	12, −67, 58	5.06
		L fusiform (19)	−24, −76, −11	5.68
		R anterior cingulate (24/32)	−21, 5, 49	4.05
		L anterior cingulate (24/32)	24, 5, 52	4.04
		R cerebellar culmen/tuber (crus 1)	33, −55, −29	4.57
		L DLPFC/middle frontal gyrus (9)	−48, −1, 34	5.10
		cerebellar vermis (6/7/8)	0, −73, −35	4.31
		R supramarginal gyrus (40)	36, −37, 46	4.80
2	403	L putamen	−21, −4, 10	4.82
		L caudate	−9, 2, 10	4.52
		L insula	−33, 14, 4	4.15
		L thalamus	−9, −1, 7	4.63
3	122	R caudate	18, 17, 7	4.05
		R putamen	21, 14, 7	3.83

*Contrast was thresholded at FDR corrected p < 0.01, extent threshold p < 0.05 (45 voxels)*.

Figure 4C shows example plots of ROIs and Table 3 gives MNI coordinates, parameter estimates and *p*-values from paired samples *t*-test for differences between sessions for each ROI. Each ROI showed an increase in activity between sessions 1 and 2, with 19 out of 24 regions showing significant increases after correction for multiple comparisons.

**Table 3:** **MNI coordinates and mean (standard error) contrast values for each region of interest for antisaccade trials session 1 and 2**.

**Region**	**MNI**	**Session 1**	**Session 2**	***p***
L dorsolateral prefrontal cortex (middle frontal gyrus, BA 9/46)	−12, 23, 34	0.81 (0.30)	1.67 (0.31)	0.065
R dorsolateral prefrontal cortex (middle frontal gyrus, BA 9/46)	54, −1, 24	0.75 (0.28)	2.54 (0.43)	0.001^*^
L ventrolateral prefrontal cortex (inferior frontal gyrus, BA 44/45/47)	−36, 11, 7,	0.82 (0.30)	2.21 (0.45)	0.011^*^
R ventrolateral prefrontal cortex (inferior frontal gyrus, BA 44/45/47)	51, −1, 22	0.72 (0.26)	1.90 (0.38)	0.014^*^
L frontal eye field (BA 6/8)	−27, −4, 58	0.65 (0.38)	5.28 (0.64)	1.26 × 10^−5^^*^
R frontal eye field (BA 6/8)	27, −13, 52	0.61 (0.20)	2.39 (0.29)	1.51 × 10^−5^^*^
Supplementary eye field (BA 6/8)	3, −1, 61	2.22 (0.45)	6.26 (0.91)	1.40 × 10^−4^^*^
L anterior cingulate (BA 24/32)	−9, 5, 52	1.31 (0.30)	3.63 (0.54)	0.001^*^
R anterior cingulate (BA 24/32)	6, 17, 40	1.62 (0.52)	3.93 (0.69)	0.014^*^
L intraparietal sulcus (BA 7/40)	−24, −55, 55	0.49 (0.40)	6.23 (0.89)	1.51 × 10^−5^^*^
R intraparietal sulcus (BA 7/40)	27, −55, 58	0.87 (0.44)	6.62 (0.98)	1.21 × 10^−5^^*^
L supramarginal gyrus (BA 40)	−42, −34, 31	0.52 (0.25)	1.94 (0.34)	0.001^*^
R supramarginal gyrus (BA 40)	27, −40, 46	0.45 (0.20)	2.60 (0.44)	0.001^*^
L precuneus (BA 7)	−18, −52, 61	1.02 (0.34)	5.01 (0.69)	9.65 × 10^−5^^*^
R precuneus (BA 7)	18, −49, 52	0.78 (0.23)	3.38 (0.41)	1.1 × 10^−5^^*^
L caudate	−21, −19, 22	0.76 (0.32)	1.26 (0.35)	0.304
R caudate	24, −16, 19	0.99 (0.27)	1.53 (0.40)	0.269
L putamen^a^	−18, −10, 7	0.36 (0.27)	2.18 (0.45)	0.001^*^
R putamen^a^	36, 11, 4	1.44 (0.37)	2.29 (0.52)	0.197
L pons	−21, −37, −38	0.42 (0.42)	1.86 (0.59)	0.023^*^
R pons	21, −34, −41	0.72 (0.41)	1.96 (0.59)	0.051
L lingual gyrus (BA 18/19)	−18, −76, −11	0.26 (0.66)	6.97 (0.94)	2.72 × 10^−5^^*^
R lingual gyrus (BA 18/19)	18, −76, −11	0.47 (0.74)	8.09 (1.08)	2.3 × 10^−5^^*^
Cerebellar vermis	6, −70, −11	1.49 (0.55)	6.56 (0.89)	1.46 × 10^−4^^*^

*MNI coordinates indicate the center of the spherical ROI. P-values are given for paired t-test for significant difference between sessions. Asterisks indicate p-values that survive correction for multiple comparisons (q = 0.0395)*.

a *Note: peak of activity is on the edge of the anatomical ROI definition*.

Figure 4D shows example scatterplots and Table 4 gives results of the correlations of each ROI with behavioral measures. In general, regions associated with antisaccade latency showed stronger positive correlations in session 2 than session 1: that is, in session 2, activity in left frontal eye fields, supplementary eye fields, left anterior cingulate, left intraparietal sulcus, bilateral precuneus, bilateral lingual gyrus and cerebellar vermis increased with increasing antisaccade latency. Activity in left caudate, right intraparietal sulcus, right supramarginal gyrus increased with increasing antisaccade latency but did not survive correction for multiple comparisons. Regions associated with antisaccade gain showed stronger negative correlations in session 2 than session 1: in other words, in session 2 activity in supplementary eye fields, left anterior cingulate, bilateral intraparietal sulcus, right supramarginal gyrus, bilateral precuneus, and bilateral lingual gyrus showed reduced activity with increasing gain. Activity in right anterior cingulate and cerebellar vermis reduced with increasing gain in session 2 but did not survive correction for multiple comparisons. For directional error rate, the increase in correlation strength between session 1 and 2 was obtained in the right supramarginal gyrus but did not survive correction for multiple comparisons.

**Table 4:** *****r***-values (***p***-values) for bivariate correlations between antisaccade regions of interest and behavioral data**.

	**Antisaccade latency**		**Directional error rate^b^**		**Saccade gain**	
**Region**	**Session 1**	**Session 2^a^**	**Session 1**	**Session 2**	**Session 1**	**Session 2^a^**
L dorsolateral prefrontal cortex	0.355 (0.048)	0.205 (0.018)				
R dorsolateral prefrontal cortex						
L ventrolateral prefrontal cortex						
R ventrolateral prefrontal cortex						
L frontal eye field	−**0.306 (0.078)**	0.612 (0.001)^*^				
R frontal eye field						
Supplementary eye field	−**0.028 (0.45)**	0.489 (0.010)^*^			−**0.04 (0.428)**	−0.604 (0.001)^*^
L anterior cingulate	−**0.136 (0.268)**	0.471 (0.013)^*^			**0.001 (0.497)**	−0.522 (0.006)^*^
R anterior cingulate					−**0.075 (0.367)**	−**0.435 (0.021)**
L intraparietal sulcus	−**0.205 (0.174)**	0.567 (0.003)^*^			**0.059 (0.395)**	−0.613 (0.001)^*^
R intraparietal sulcus	−**0.112 (0.305)**	**0.383 (0.039)**			−**0.137 (0.266)**	−0.55 (0.004)^*^
L supramarginal gyrus						
R supramarginal gyrus	−**0.078 (0.361)**	**0.389 (0.037)**	**0.292 (0.088)**	−**0.414 (0.028)**	−**0.001 (0.498)**	−0.598 (0.002)^*^
L precuneus	−**0.198 (0.182)**	0.474 (0.013)^*^			**0.037 (0.433)**	−0.625 (0.001)^*^
R precuneus	−**0.217 (0.160)**	0.527 (0.006)^*^			**0.014 (0.475)**	−0.501 (0.009)^*^
L caudate	−**0.359 (0.046)**	**0.082 (0.358)**				
R caudate						
L putamen	0.357 (0.047)	0.161 (0.237)				
R putamen						
L pons						
R pons						
L lingual gyrus	**0.008 (0.486)**	0.535 (0.005)^*^			**0.344 (0.054)**	−0.526 (0.006)^*^
R lingual gyrus	0.086 (0.349)	0.573 (0.003)^*^			**0.026 (0.452)**	−0.498 (0.009)^*^
Cerebellar vermis	0.273 (0.104)	0.495 (0.010)^*^			**0.015 (0.473)**	−**0.415 (0.027)**

*Values are given only for regions showing at least 1 correlation with the behavioral measure p < 0.05 in either session. Asterisks indicate values that survived correction for multiple comparisons. Bold values indicate regions showing significant change in correlation strength between sessions 1 and 2, tested using confidence intervals (Zou, 2007)*.

*Note*:

a *both q = 0.0173*.

b *All analyses excluded the antisaccade directional error rate outlier*.

## Discussion

In this study we report an association between ocular motor behavior and change in activation levels of neural regions implicated in ocular motor control as a function of antisaccade training. Extended training on the antisaccade task results in changes in saccade latency and gain, and increases in activity across the extended ocular motor network. Following 2 weeks of training on an antisaccade task optimized to induce learning effects, antisaccade latency and gain decreased relative to pre-training levels. Training was associated with *increased* activity in the ocular motor network relative to pre-training levels. In session 2, larger antisaccade latency was associated with larger activity in fronto-parietal-cerebellar ocular motor regions, and smaller antisaccade gain was associated with larger activity in fronto-parietal ocular motor regions.

Participants performed antisaccade trials faster, with no change in performance accuracy (directional error rates), following 2 weeks of training. Interestingly, it appears that this improvement in saccade latency came at the cost of spatial accuracy, with antisaccade trials becoming more hypometric in session 2 compared to session 1. Previous behavioral effects of extended training have been equivocal, with one reporting no change in behavior (Lee et al., 2012), one reporting reduced directional error rates with no change in latency (Dyckman and McDowell, 2005; antisaccade training group), and another reporting reduced directional error rates and reduction in latency (Unsworth et al., 2011). The inconsistent previous results may be attributable to these studies using a different paradigm for the training vs. test sessions, with the training paradigms requiring a button press response in addition to an eye movement, and test paradigms requiring an ocular motor response only. Importantly, even in our training paradigm that was optimized for learning effects (identical training and test paradigms, interleaved presentation of antisaccade and prosaccade trials; see Lin et al., 2013), we found that antisaccade trials showed larger latency and directional error rate than prosaccade trials even after 2 weeks of training, suggesting that the antisaccade response remained effortful, and did not become automatic even after extended training.

To our knowledge, only one previous study has examined change in saccade gain following repeated testing. Ettinger et al. (2003) reported a shift in saccade gain of the initial saccade from hyper- to hypometric following repeat exposure to the task. We revealed a decrease in gain following training for both antisaccade and prosaccade trials^2^. Why gain should become more hypometric following training is not clear. However, given that latencies were significantly reduced following training it may be that once the antisaccade stimulus-response relationship is established, hypometricity represents a speed-spatial accuracy trade-off. Seemingly, as long as a saccade to the suddenly appearing non-target stimulus is suppressed, and a saccade is generated in the appropriate direction (i.e., appropriate stimulus-response relationship), the spatial accuracy of the first saccade may become relatively unimportant if the end point accuracy is achievable with subsequent saccades. Specifically, the behavioral goal shifts from that of performing spatially accurate saccades to maintaining the stimulus-response relationship required by the task.

Another contributing factor to the hypometricity of saccades in both pro- and antisaccades is likely to be psychological fatigue. Our procedure involved training participants to perform a large numbers of repetitive saccades from central fixation point toward a fixed-distance peripheral target, regardless of condition, without trial-to-trial reward. Based on findings by Prsa et al. (2010), reduced gain over time during repetitive saccadic movements is unrelated to oculo-muscular fatigue, but may arise from neuronal habituation to the consequences of a repetitive visual stimulus. Diminished attention, motivation and alertness may reduce top-down input to the superior colliculus from higher cortical areas, can result in reductions in saccade amplitude over time.

Training on the antisaccade task resulted in increases in fMRI activity in the brain, both at a whole brain level and across the fronto-basal ganglia-parietal-cerebellar ocular motor regions of interest. While the effect was obtained consistently across the brain, the effect appeared to be largest in the frontal and supplementary eye field, intraparietal sulcus, lingual gyrus and cerebellar vermis. The frontal and supplementary eye fields, intraparietal sulcus and cerebellar vermis play a central role in ocular motor control, and neurophysiological studies have shown these areas are the primary locations of saccade neurons in the brain (reviewed in Krauzlis, 2005; Figure 1). Neurons in the frontal eye fields determine when voluntary eye movements are initiated (Sato and Schall, 2003), and are particularly involved in saccades to remembered locations (Dias et al., 1995). The supplementary eye fields are important for internally-generated saccades, and it is thought to be the initiating locus of the signal to inhibit the prepotent prosaccade response (Schlag-Rey et al., 1997). The intraparietal sulcus is crucial to the process of vector inversion, the translation of the visual location of the target to the mirror image location (Pierrot-Deseilligny et al., 2004; Medendorp et al., 2005), and the cerebellar vermis is crucial to the determination of the final eye position of the saccade (related to saccade gain; Thier et al., 2002). The finding that the lingual gyrus in particular showed increased activity following antisaccade training is interesting, as this region is commonly reported in fMRI studies of antisaccades (Jamadar et al., 2013) but is not classically linked to ocular motor control. The lingual gyrus is involved in color processing (Miceli et al., 2001) and may be involved in visuo-spatial learning and memory (Bogousslavsky et al., 1987), suggesting that the increased activity in this region following antisaccade training may be linked to learning of visuo-spatial representations of the color cue-task mapping used in our design.

Our finding of an increase in neural activity following training is in contrast to the only other fMRI study of cognitive training on the antisaccade task. Lee et al. (2012) found a decreased number of activated voxels in the frontal and supplementary eye fields, superior parietal lobule and cuneus following 1 week of training on a button-press antisaccade task. Pre- vs. post-training activation amplitude differences were obtained in that study but are hard to evaluate as many significant differences were obtained in time-bins outside of active task performance (their Figures 7–10). Importantly, that study did not find any effects of training on behavioral measures of antisaccade performance, despite finding performance improvements on the button-press training task across the training sessions. This suggests that performance improvements seen in the practice sessions using the button-press task did not transfer to the saccade test task. Previous studies of transfer of cognitive training improvements suggest that acquired skills are specific to the practiced task, or to tasks that rely on almost identical processes and networks (Dahlin et al., 2008; Jolles et al., 2010). It has not been established how the button-press antisaccade task maps onto the processing and neural resources required by the antisaccade task, and the variable effects of training on the antisaccade task reported previously (Dyckman and McDowell, 2005; Unsworth et al., 2011; Lee et al., 2012) suggests that the tasks may not be similar enough so as to induce transfer of cognitive training.

fMRI activity in the ocular motor network became significantly correlated with behavior following antisaccade training. Antisaccade latency became significantly positively associated with fMRI activity in left frontal eye field, supplementary eye field, left anterior cingulate, left intraparietal sulcus, bilateral precuneus, and left lingual gyrus following training. (Right intraparietal sulcus and supramarginal gyrus were more strongly correlated with latency in session 2 but did not survive correction for multiple comparisons; left dorsolateral prefrontal cortex, left putamen, right lingual gyrus and cerebellar vermis also showed a trend toward stronger association with latency but the change in *r*-value was not significant). So, individuals with longer latency showed increased activity in these regions (conversely, individuals with faster latency showed decreased activity in the ocular motor network following training). Antisaccade gain became significantly negatively associated with fMRI activity in the supplementary eye fields, bilateral anterior cingulate, bilateral intraparietal sulcus, right supramarginal gyrus, bilateral precuneus, bilateral lingual gyrus and cerebellar vermis following training. Inspection of Figure 4D shows that individuals showing hypometricity in session 2 showed larger activity in the ocular motor network than those showing more accurate (saccade gain ~= 1.0) saccades. Together, these results suggest that comparatively worse performance (i.e., relative to other individuals) in session 2 was associated with increased fMRI activity in the ocular motor network.

Generally, the neural effects of cognitive training are quite variable, with some showing decreases in activity following training, others showing increases in activity, and others showing a pattern of increases in some regions and decreases in others (Kelly and Garavan, 2005). A finding of decreased activity in task-related regions following training is the most common finding, although our finding that activation increased in the ocular motor network following training is consistent with a large number of studies of cognitive training. In an attempt to consolidate the variability in the cognitive training literature, Kelly and Garavan (2005) argue that training of high-level cognitive skills requiring prefrontal cortex involvement is more likely to result in activation decreases following training, whereas training of sensory and motor tasks is more likely to result in activation increases following training. While the antisaccade task at its most fundamental level centers upon a motor response, the strong requirement for top-down inhibition of the prepotent response and the processes of vector inversion suggest that the more likely effect should have been a decrease in activity following training. Even in their formulation of cognitive training effects, Kelly and Garavan conceded that the apparent “training-related decrease in activity for controlled tasks/increase in activity for motor tasks” dichotomy is too simplistic, and numerous exceptions exist (e.g., Jolles et al., 2010 obtained an increase in prefrontal activity following working memory training; Erickson et al., 2007 obtained an increase in the dorsolateral prefrontal cortex following dual-task training). Kelly and Garavan note that exceptions to the dichotomy are most likely to occur when performance does not reach automaticity. Our results suggest a refinement of this dichotomy: activity is enhanced in the task-relevant network for poorer performing subjects (slower latency and more hypometric saccade gain) than better performing subjects following training.

### Strengths, limitations, and directions for future research

This study represents the most robust test of antisaccade training to date, using a training paradigm optimized to induce learning, high-resolution fMRI and robust correction for multiple comparisons. One limitation of this study is that we did not monitor training sessions. Although we are confident that the subjects engaged fully in the training process, we did rely on self-report of the number of completed training sessions completed.

We have argued here that button-press antisaccade tasks are likely to introduce different cognitive processes and cognitive demands compared to the classic ocular motor antisaccade task. We feel that this argument is a non-sequitur, as the inclusion of a button press at the very least introduces additional activity in motor cortico-basal ganglia-cortical loops, and paradigms that introduce a stimulus discrimination task [such as the “T” orientation task used by Dyckman and McDowell (2005) and Lee et al. (2012); and letter discrimination task used by Unsworth et al. (2011)] introduce additional stimulus-response demands. No study to date has tested the concordance of antisaccade results obtained between paradigms requiring a button-press response and those requiring only an eye movement, however evidence from other paradigms comparing saccade responses to pointing movements show differences in activity in motor, premotor and occipital regions (Connolly et al., 2007; Hagler et al., 2007). Future studies should address this issue in the antisaccade task.

One final question that remains is whether antisaccade performance can ever become as automatic as a prosaccade response. Given that saccadic eye movement toward a peripheral stimulus (i.e., prosaccade response) is one of the most prepotent responses in our behavioral repertoire, it is possible that automaticity could never be obtained for the antisaccade response, even with very long training paradigms. Here, participants completed a total of 892 antisaccade trials over the course of 14 days, and performance did not approach that of prosaccade trials (Figure 3). Previous studies that used the button-press training paradigm also did not find antisaccade performance approach prosaccade performance levels with 982 trials (Lee et al., 2012) or 1900 trials (Dyckman and McDowell, 2005). The exception to this is Unsworth et al. (2011), who found that following 3500 antisaccade trials, antisaccade performance did not significantly differ from prosaccade performance. An important caveat is that this was assessed by comparing performance on trials 3250–3500 of the antisaccade training group to performance of the sole 250 trials completed by the prosaccade group in a between-subjects design. As shown here, our results question the validity of this comparison, as prosaccade performance and fMRI activity were amenable to cognitive training (see Supplementary Material). It therefore remains to be determined if antisaccade performance can be equated with prosaccade performance following extensive training.

## Conclusions

Our results suggest that individuals that show comparatively worse antisaccade performance following 2 weeks of antisaccade training show larger fMRI activity in the ocular motor network. Importantly, changes in fMRI activity following training that were associated with behavioral change occurred primarily in cortical, rather than basal ganglia, ocular motor regions. In general, cortical ocular motor regions are involved in the cognitive aspects of the saccade response, including endogenous preparation of the saccade, inhibition of the competing prosaccade response, and translation of the visual location of the target (exogenous information) to the mirror image location (endogenous task goal; Ford et al., 2005; Medendorp et al., 2005; Brown et al., 2006; Ettinger et al., 2008). In contrast, subcortical regions such as the basal ganglia are more involved in mediating response threshold and bias for antisaccade and prosaccade responses across trials (Isoda and Hikosaka, 2011).

## Ethical approval

All procedures performed in studies involving human participants were in accordance with the ethical standards of the institutional and/or national research committee and with the 1964 Helsinki declaration and its later amendments or comparable ethical standards. Informed consent was obtained from all individual participants included in the study.

## Author contributions

SJ, BJ, GE, and JF designed the study. SJ and BJ collected the data. BJ and MC conducted the ocular motor data processing. SJ conducted the behavioral and fMRI analyses. SJ wrote the paper with edits by JF, BJ, and GE. All authors approve the final version for publication.

## Funding

This work was supported by an Australian Research Council Discovery Grant DP110102084 (JF and GE). GE is a National Health and Medical Research Committee Principal Research Fellow. SJ is supported by an Australian Research Council Discovery Early Career Researcher Award DE150100406.

### Conflict of interest statement

The authors declare that the research was conducted in the absence of any commercial or financial relationships that could be construed as a potential conflict of interest.

## Footnotes

^1^This result remained when removing the cluster threshold (i.e., voxel-wise FDR *q* < 0.01, *k* = 0 voxels). No region showed significant activity in baseline > antisaccade averaged over session.

^2^Unlike the present study, Ettinger et al. (2003) also found a decrease in directional error rate (21 vs. 16%) on retest. It is unclear why we did not obtain an effect of training on error rate, however our participants were highly accurate in the first session (~90% accuracy), which may represent a ceiling effect.
