# Supplementary material for: Behavioral and Neural Plasticity of Ocular Motor Control: Changes in Performance and fMRI Activity Following Antisaccade Training
Source: Front Hum Neurosci. 2015 Dec 18;9:653. doi: 10.3389/fnhum.2015.00653 (PMC4683540; doi:10.3389/fnhum.2015.00653)
Supplement: Supplementary file 1 [file SupplementaryMaterial.DOCX]

***Behavioural and neural plasticity of ocular motor control: changes in performance and fMRI activity following antisaccade training***

S.D. Jamadar, B. Johnson, M. Clough, G.F. Egan, J. Fielding

**Supplementary Material:**

**Effect of Training on Prosaccade Performance**

The primary focus of the current study was the effects of training on antisaccade performance, however subjects were also tested and trained on prosaccade trials. Here, we present fMRI results for the prosaccade trials.

**Methods**

Methods for the prosaccade analysis were identical to that of the antisaccade analysis, with exceptions noted here.

Contrast images for prosaccade > baseline averaged over session and prosaccade session 2 > session 1 were entered into a second-level random effects analysis and thresholded at voxel-wise FDR corrected p < .01, cluster extent threshold uncorrected p<.05. Regions of interest were defined by using the same anatomical masks of ocular motor regions used in the antisaccade analysis to inclusively mask the prosaccade > baseline comparison (averaged over session) and thresholded at voxel-wise FDR corrected p < .01. The coordinate of maximum activity within the mask was selected and a spherical ROI (10mm) was created around this peak of activity. Table S3 gives MNI coordinates for each ROI. MarsBaR (v0.43, Brett et al., 2002) was used to extract parameter estimates (contrast values) for the prosaccade greater than baseline comparison from each of the ROIs for each individual and session separately. Correlations were conducted between each ROI and prosaccade latency, directional error rate and gain for each session separately.

**Results**

Figure S1A shows whole brain fMRI results and Table S1 gives region labels, peak MNI and t-values for the prosaccade compared to baseline comparison, averaged over session. Prosaccade trials activated a bilateral fronto-basal ganglia-parieto-cerebellar network consistent with previous studies (Jamadar et al., 2013). Figure S1B shows fMRI results and Table S2 gives region labels, peak MNI and t-values for the effect of session for prosaccade compared to baseline comparison. Prosaccade trials showed increased activity in many of the regions in the prosaccade network in session 2 compared to session 1. No region showed larger activity in session 1 compared to session 2.

Figure S1C shows example plots of ROIs and Table S3 gives MNI coordinates, parameter estimates and p values from paired samples t-test for differences between sessions for each ROI. Each ROI showed an increase in activity between sessions 1 and 2, with 15 of 24 regions showing significant increases after correction for multiple comparisons.

Figure S1D shows example scatterplots and Table S4 gives the results of the correlations of each ROI with behavioural measures. Only saccade gain showed significant relationship with fMRI activity after correction for multiple comparisons. Increased activity in left frontal eye field, left anterior cingulate, right supramarginal gyrus, right caudate, right putamen, right pons and cerebellar vermis was associated with increased gain in session 2 but not session 1. A similar relationship was obtained for right intraparietal sulcus, left caudate, left putamen, and bilateral lingual gyrus but these did not survive correction for multiple comparisons. Activity in a number of regions showed relationships with prosaccade directional error rate and latency but these did not survive correction for multiple comparisons.

**Discussion**

Latency and saccade decreased and fMRI activity in the ocular motor network increased following two weeks of prosaccade training. Prosaccade latency and directional error rate showed some changes in correlation strength prior to vs. after training, however none of these correlations reached significance after correction for multiple comparisons. fMRI activity in left frontal eye field, left anterior cingulate, right supramarginal gyrus, right caudate, right putamen, right pons and cerebellar vermis became significantly positively associated with saccade gain following prosaccade training. Examining Figure S1 indicates that fMRI activity in these regions increased as saccade gain became less hypometric (indeed, some individuals are hypermetric) in session 2.

These results clearly demonstrate that prosaccade performance is susceptible to change with extended practice, and that these performance changes are associated with significant changes in activity across the ocular motor network. In their examination of antisaccade performance changes with practice, Unsworth et al. (2011) compared antisaccade performance on trials 3250-3500 to prosaccade performance on the sole 250 prosaccade trials performed by the prosaccade group in a between-subjects design. Our results clearly demonstrate that this comparison is not suitable for examining whether antisaccade performance becomes automatic, as the prepotent prosaccade response and its associated neural activity is also changed following extensive practice.

Interestingly, training increased activity in the ocular motor network for prosaccade trials as well as antisaccade trials. Kelly and Garavan (2005) argued that simple motor tasks are more likely to show increased activity in task-relevant regions following training, and these results are compatible with this argument. Unlike antisaccade trials, where the increased ocular motor network activity was associated with poorer performance, for prosaccade trials the association between changes in activity and performance is less straight-forward, as a few individuals showed a tendency towards hypermetricity following training.


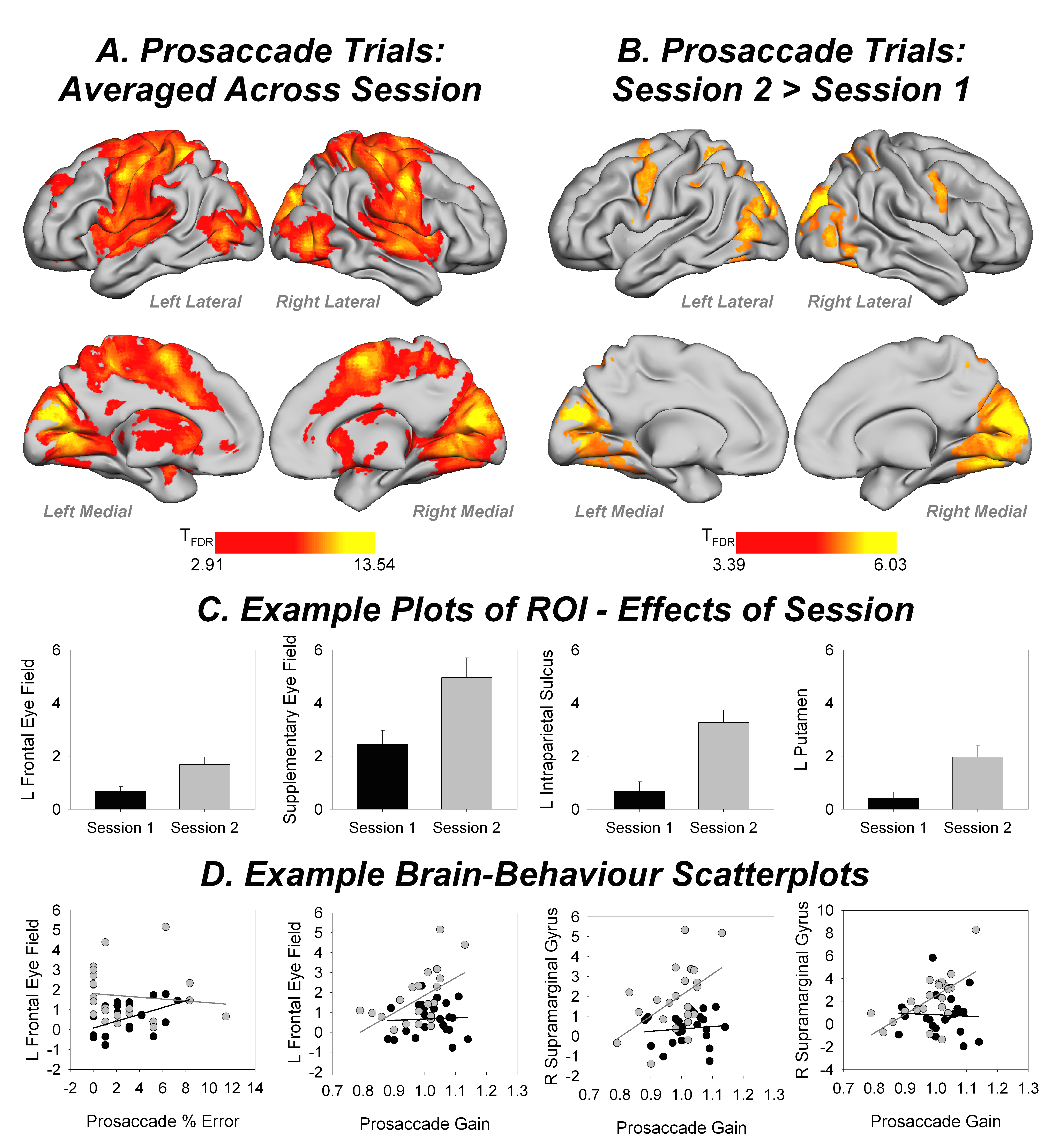


**Fig S1:** A. fMRI activity for the prosaccade vs. baseline condition, averaged over session. Contrast thresholded at FDR corrected p < .01, extent threshold p < .05. B. fMRI activity (parameter estimates) for prosaccade session 2 > session 1. Contrast thresholded at FDR corrected p < .01, extent threshold p < .05. C. Example scatterplots for effects of session (parameter estimates) in four regions of interest (ROIs). Error bars show standard error. D. Example scatterplots for brain-behaviour correlations for session 1 (parameter estimates; black) and session 2 (white). Abbreviations: L: left; R: right

**Table S1:** Peak MNI coordinates, t-values and region labels for the prosaccade > baseline whole brain contrast, averaged over session. Contrast was thresholded at FDR corrected p<.01, extent threshold p<.05 (70 voxels).

| Cluster # | # Voxels | Region Label (BA) | Peak MNI | T value |
| --- | --- | --- | --- | --- |
| 1 | 23797 | L Cuneus (19) | -27 -73 16 | 13.54 |
|  |  | R Frontal Eye Field/Precentral Gyrus (6) | 12 -1 55 | 7.08 |
|  |  | R Superior Temporal Gyrus (22/41) | -54 2 -7 | 7.28 |
|  |  | R Lingual Gyrus (18) | 6 -73 1 | 5.48 |
|  |  | Supplementary Eye Field | 0 -1 61 | 8.09 |
|  |  | L Middle Occipital Gyrus (18/19) | -18 -88 16 | 7.41 |
|  |  | L Superior Temporal Gyrus (22/41) | 63 -10 -2 | 7.21 |
|  |  | L Lingual Gyrus (18) | -6 -73 1 | 8.19 |
|  |  | R Cuneus (18/19) | 6 -82 22 | 10.33 |
|  |  | L Precuneus (7) | -18 -46 55 | 8.73 |
|  |  | L IPS/Superior Parietal Lobule (7) | -24 -52 58 | 7.19 |
|  |  | L Cerebellar Culmen/Declive (Lobule 6) | -21 -64 -23 | 5.22 |
|  |  | R Cerebellar Culmen/Declive (Lobule 6) | 27 -64 -26 | 4.79 |
|  |  | L Insula | -36 -10 1 | 5.83 |
|  |  | R IPS/Superior Parietal Lobule (7) | 27 -52 58 | 5.10 |
|  |  | L Superior Occipital Gyrus (19) | -18 -82 31 | 10.21 |
|  |  | R Insula | 33 11 7 | 7.00 |
|  |  | R Superior Occipital Gyrus (19) | 18 -85 28 | 9.32 |
|  |  | R Middle Occipital Gyrus (18/19) | 39 -67 4 | 7.41 |
|  |  | R Putamen | 30 -16 -2 | 7.87 |
|  |  | L Putamen | -18 -10 10 | 7.81 |
|  |  | L Anterior Cingulate (24/32) | -18 -7 46 | 7.45 |
|  |  | L DLPFC/Middle Frontal Gyrus (9) | -21 29 31 | 4.78 |
|  |  | R Fusiform (19) | 36 -61 -11 | 5.26 |
|  |  | L Cerebellar Culmen/Declive (Lobule 8) | -24 -55 -29 | 3.76 |
|  |  | R Precuneus (7) | 18 -52 58 | 8.20 |
|  |  | R Supramarginal Gyrus (40) | 27 -40 46 | 6.38 |
|  |  | R Middle Temporal Gyrus (19/39) | 42 -67 4 | 7.89 |
|  |  | R Anterior Cingulate (24/32) | 15 -1 49 | 6.11 |
|  |  | L Caudate | -18 14 4 | 5.57 |
|  |  | L Supramarginal Gyrus (40) | -48 -25 13 | 5.55 |
|  |  | R Cerebellar Declive/Culmen/Tonsil (Crus 1) | 42 -49 -35 | 3.97 |
|  |  | L Thalamus | -15 -10 10 | 5.75 |
|  |  | R Cerebellar Culmen/Declive (Lobule 8) | 30 -55 -47 | 4.24 |
|  |  | R Inferior Occipital Gyrus (19) | 45 -76 -2 | 7.04 |
|  |  | L Cerebellar Culmen (Lobule 4/5) | -9 -55 -11 | 5.34 |
|  |  | R Caudate | 21 -4 16 | 5.96 |
|  |  | R DLPFC/Middle Frontal Gyrus (9) | 15 29 31 | 4.12 |
|  |  | Cerebellar Vermis (Lobule 4/5/6) | 6 -61 -5 | 8.13 |
|  |  | R Thalamus | 18 -16 10 | 5.68 |
|  |  | L Fusiform (19) | -24 -73 -14 | 4.37 |
|  |  | R Cerebellar Culmen (Lobule 4/5) | 12 -55 -11 | 5.26 |
|  |  | L VLPFC/Inferior Frontal Gyrus (44) | -60 5 13 | 7.04 |
|  |  | R VLPFC/Inferior Frontal Gyrus (44) | 51 -4 22 | 7.09 |

**Table S2:** Peak MNI coordinates, t-values and region labels for the prosaccade session 2 > session 1 whole brain contrast. Contrast was thresholded at FDR corrected p<.01, extent threshold p<.05 (40 voxels).

| Cluster # | # Voxels | Region Label (BA) | Peak MNI | T value |
| --- | --- | --- | --- | --- |
| 1 | 6051 | R Cuneus (19) | 3 -82 22 | 6.30 |
|  |  | R Lingual Gyrus (18) | 6 -79 -8 | 5.92 |
|  |  | L Middle Occipital Gyrus | -27 -85 28 | 5.53 |
|  |  | L Lingual Gyrus | -21 -73 -11 | 5.33 |
|  |  | R Middle Occipital Gyrus | 30 -82 28 | 5.37 |
|  |  | L Cerebellar Lobule 6 | -15 -73 -17 | 4.42 |
|  |  | L Cuneus | -15 -85 31 | 4.34 |
|  |  | R Superior Occipital Gyrus | 15 -97 16 | 6.04 |
|  |  | R Cerebellar Lobule 6 | 33 -58 -23 | 4.34 |
|  |  | L IPS/Superior Parietal Lobule (7) | -24 -52 55 | 4.65 |
|  |  | L Superior Occipital Gyrus | -21 -82 31 | 5.56 |
|  |  | R Fusiform | 33 -79 -17 | 5.55 |
|  |  | L Fusiform | -24 -73 -14 | 5.60 |
|  |  | L Cerebellar Crus 1 | -18 -76 -23 | 3.51 |
|  |  | R Middle Temporal Gyrus | 48 -73 16 | 4.17 |
|  |  | R Inferior Occipital Gyrus | 30 -82 -14 | 4.95 |
|  |  | L Precuneus | -12 -67 58 | 3.86 |
|  |  | L Inferior Occipital Gyrus | -42 -70 -14 | 3.89 |
|  |  | R Cerebellar Crus 1 | 24 -79 -23 | 3.45 |
|  |  | Cerebellar Vermis (4/5/6) | 6 -73 -11 | 4.86 |
| 2 | 762 | L Frontal Eye Field/Precentral Gyrus (6) | -27 -1 61 | 4.61 |
|  |  | R Supplementary Eye Field | 3 -1 61 | 3.38 |
|  |  | L DLPFC/Middle Frontal Gyrus (9/44) | -27 -1 61 | 4.62 |
|  |  | L Thalamus | -12 -19 -2 | 4.07 |
|  |  | L Caudate | -9 2 10 | 3.56 |
| 3 | 71 | L Putamen | -21 -4 10 | 3.72 |
|  |  | R Caudate | 21 17 7 | 3.56 |
|  |  | R Putamen | 21 14 7 | 3.70 |
| 4 | 386 | R DLPFC/Middle Frontal Gyrus (9) | 54 2 34 | 4.26 |
|  |  | R Frontal Eye Field/Precentral Gyrus (6) | 27 -7 58 | 3.67 |
| 5 | 422 | R IPS/Superior Parietal Lobule (7) | 27 -52 58 | 4.65 |
|  |  | R Precuneus (7) | 12 -67 61 | 4.14 |
|  |  | R Supramarginal Gyrus (40) | 30 -40 43 | 4.18 |

**Table S3:** MNI coordinates and mean (standard error) contrast values for each prosaccade region of interest for session 1 and 2. MNI coordinates indicate the centre of the spherical ROI. P values are given for paired t-test for significant difference between sessions. Asterisks indicate p values that survive correction for multiple comparisons (q=.0313)

| ***Region*** | ***MNI*** | ***Session 1*** | ***Session 2*** | ***p*** |
| --- | --- | --- | --- | --- |
| L Dorsolateral Prefrontal Cortex | -21 29 31 | .56 (.31) | 1.16 (.24) | .185 |
| R Dorsolateral Prefrontal Cortex | 15 29 31 | .47 (.27) | .87 (.21) | .297 |
| L Ventrolateral Prefrontal Cortex | -60 5 13 | .70 (.33) | 2.59 (.45) | .005* |
| R Ventrolateral Prefrontal Cortex | 51 -4 22 | 1.13 (.30) | 2.45 (.40) | .013* |
| L Frontal Eye Field | -21 -7 46 | .67 (.17) | 1.69 (.29) | .010* |
| R Frontal Eye Field | 12 -1 55 | 1.21 (.29) | 2.74 (.49) | .016* |
| Supplementary Eye Field | 0 -1 61 | 2.44 (.54) | 4.96 (.74) | .009* |
| L Anterior Cingulate | -18 -7 46 | .71 (.16) | 1.51 (.28) | .035 |
| R Anterior Cingulate | 15 -1 49 | .83 (.24) | 1.83 (.37) | .043 |
| L Intraparietal Sulcus | -24 -52 58 | .68 (.35) | 3.27 (.47) | 1.83 x 10^-4^* |
| R Intraparietal Sulcus | 27 -52 58 | .67 (.37) | 3.41 (.60) | 3.97 x 10^-4^* |
| L Supramarginal Gyrus | -48 -25 13 | 2.26 (.46) | 2.94 (.65) | .313 |
| R Supramarginal Gyrus | 27 -40 46 | .39 (.16) | 1.86 (.35) | 2.14 x 10^-4^* |
| L Precuneus | -18 -46 55 | .88 (.26) | 1.96 (.30) | .021* |
| R Precuneus | 18 -52 58 | .88 (.31) | 3.16 (.42) | 2.73 x 10^-4^* |
| L Caudate | -18 14 4 | 1.02 (.41) | 2.24 (.47) | .073 |
| R Caudate | 21 -4 16 | .79 (.36) | 2.18 (.45) | .029* |
| L Putamen | -18 -10 10 | .41 (.23) | 1.97 (.43) | .008* |
| R Putamen | 30 -16 -2 | .53 (.32) | 1.39 (.42) | .210 |
| L Pons | -21 -40 -35 | .54 (.43) | 1.58 (.61) | .077 |
| R Pons | 24 -43 -38 | .74 (.35) | 1.90 (.60) | .062 |
| L Lingual Gyrus | -6 -73 1 | 2.08 (.85) | 7.83 (1.16) | .001* |
| R Lingual Gyrus | 6 -73 1 | 1.91 (.91) | 8.36 (1.14) | 2.83 x 10^-4^* |
| Cerebellar Vermis | 6 -61 -5 | 1.67 (.50) | 4.93 (.66) | .001* |

**Table S4:** r values (p values) for bivariate correlations between prosaccade regions of interest and behavioural data. Values are given only for regions showing at least 1 correlation with the behavioural measure p<.05 in either session. Asterisks indicate values that survived correction for multiple comparisons. Bold values indicate regions showing significant change in correlation strength between sessions 1 and 2, tested using confidence intervals (Zou, 2006).

|  | ***Prosaccade Latency*** | | ***Directional Error Rate*** | | ***Saccade Gain*** | |
| --- | --- | --- | --- | --- | --- | --- |
| ***Region*** | ***Session 1*** | ***Session 2*** | ***Session 1*** | ***Session 2*** | ***Session 1*** | ***Session 2^a^*** |
| L Dorsolateral Prefrontal Cortex |  |  | **.380 (.037)** | **.021 (.464)** |  |  |
| R Dorsolateral Prefrontal Cortex |  |  |  |  |  |  |
| L Ventrolateral Prefrontal Cortex |  |  |  |  |  |  |
| R Ventrolateral Prefrontal Cortex |  |  |  |  |  |  |
| L Frontal Eye Field |  |  | **.492 (.008)** | **-.114 (.306)** | **.052 (.406)** | **.527 (.006)*** |
| R Frontal Eye Field |  |  |  |  |  |  |
| Supplementary Eye Field |  |  |  |  |  |  |
| L Anterior Cingulate |  |  | **.513 (.006)** | **-.056 (.401)** | **.070 (.375)** | **.545 (.004)*** |
| R Anterior Cingulate |  |  |  |  |  |  |
| L Intraparietal Sulcus |  |  |  |  | .282 (.096) | .412 (.028) |
| R Intraparietal Sulcus |  |  |  |  | **.097 (.329)** | **.394 (.035)** |
| L Supramarginal Gyrus |  |  | **.448 (.016)** | **.072 (.376)** |  |  |
| R Supramarginal Gyrus |  |  | **.358 (.047)** | **-.076 (.368)** | **.111 (.308)** | **.509 (.008)*** |
| L Precuneus |  |  | **.368 (.042)** | **-.133 (.342)** |  |  |
| R Precuneus |  |  |  |  |  |  |
| L Caudate | **.364 (.044)** | **-.314 (.077)** |  |  | **-.418 (.024)** | **.187 (.203)** |
| R Caudate |  |  |  |  | **-.051 (.409)** | **.619 (.001)*** |
| L Putamen |  |  |  |  | **-.130 (.278)** | **.364 (.048)** |
| R Putamen | **.404 (.028)** | **-.275 (.107)** | **.383 (.036)** | **.068 (.381)** | **-.231 (.144)** | **.488 (.011)*** |
| L Pons |  |  | **.384 (.035)** | **-.19 (.199)** | .259 (.116) | .446 (.019) |
| R Pons |  |  | **.404 (.028)** | **-.092 (.342)** | **.203 (.177)** | **.685 (2.19 x 10^-4^)*** |
| L Lingual Gyrus |  |  |  |  | **-.122 (.29)** | **.372 (.044)** |
| R Lingual Gyrus |  |  | **.429 (.021)** | **-.001 (.498)** | **-.136 (.267)** | **.374 (.043)** |
| Cerebellar Vermis |  |  | **.499 (.008)** | **.124 (.291)** | **.149 (.249)** | **.549 (.004)*** |

Notes: ^a^ q=.0146

**Supplementary Material:**

**Ocular Motor Error Data**

|  | **Session 1** | | | | **Session 2** | | | |
| --- | --- | --- | --- | --- | --- | --- | --- | --- |
|  | **Minimum** | **Maximum** | **Mean** | **Standard Error** | **Minimum** | **Maximum** | **Mean** | **Standard Error** |
| Prosaccade |  |  |  |  |  |  |  |  |
| Directional Error | 0 | 8.33 | 3.44 | .50 | 0 | 11.45 | 2.62 | .69 |
| Anticipatory Error | 0 | 12.50 | 1.27 | .62 | 0 | 6.25 | 1.00 | .34 |
| Directional Anticipatory Error | 0 | 12.50 | 4.71 | .68 | 0 | 11.45 | 3.62 | .78 |
| Undefined | 0 | 4.17 | .68 | .20 | 0 | 4.17 | .54 | .23 |
| Antisaccade |  |  |  |  |  |  |  |  |
| Directional Error | 2.08 | 39.58 | 10.69 | 1.90 | 0 | 39.58 | 10.32 | 2.44 |
| Anticipatory Error | 0 | 4.17 | .59 | .24 | 0 | 1.04 | .14 | .07 |
| Directional Anticipatory Error | 2.08 | 43.75 | 11.28 | 2.09 | 0 | 40.63 | 10.46 | 2.46 |
| Undefined | 0 | 2.08 | .41 | .14 | 0 | 3.13 | .22 | .15 |
| Blink | 0 | 10.94 | 1.95 | .59 | 0 | 1.56 | .32 | .09 |
| Did not look | 0 | 1.56 | .07 | .07 | 0 | 0 | 0 | 0 |
| Unstable baseline | 0 | 3.64 | .79 | .26 | 0 | 1.04 | .09 | .05 |
| Small saccade | 0 | 0 | 0 | 0 | 0 | 0 | 0 | 0 |
| Signal dropout | 0 | 8.33 | 1.70 | .56 | 0 | 20.3 | 3.03 | 1.09 |

**Supplementary Material:**

**Brain-Behaviour Scatterplots**

Scatterplots of all relationships reported in Table 4 (main text).


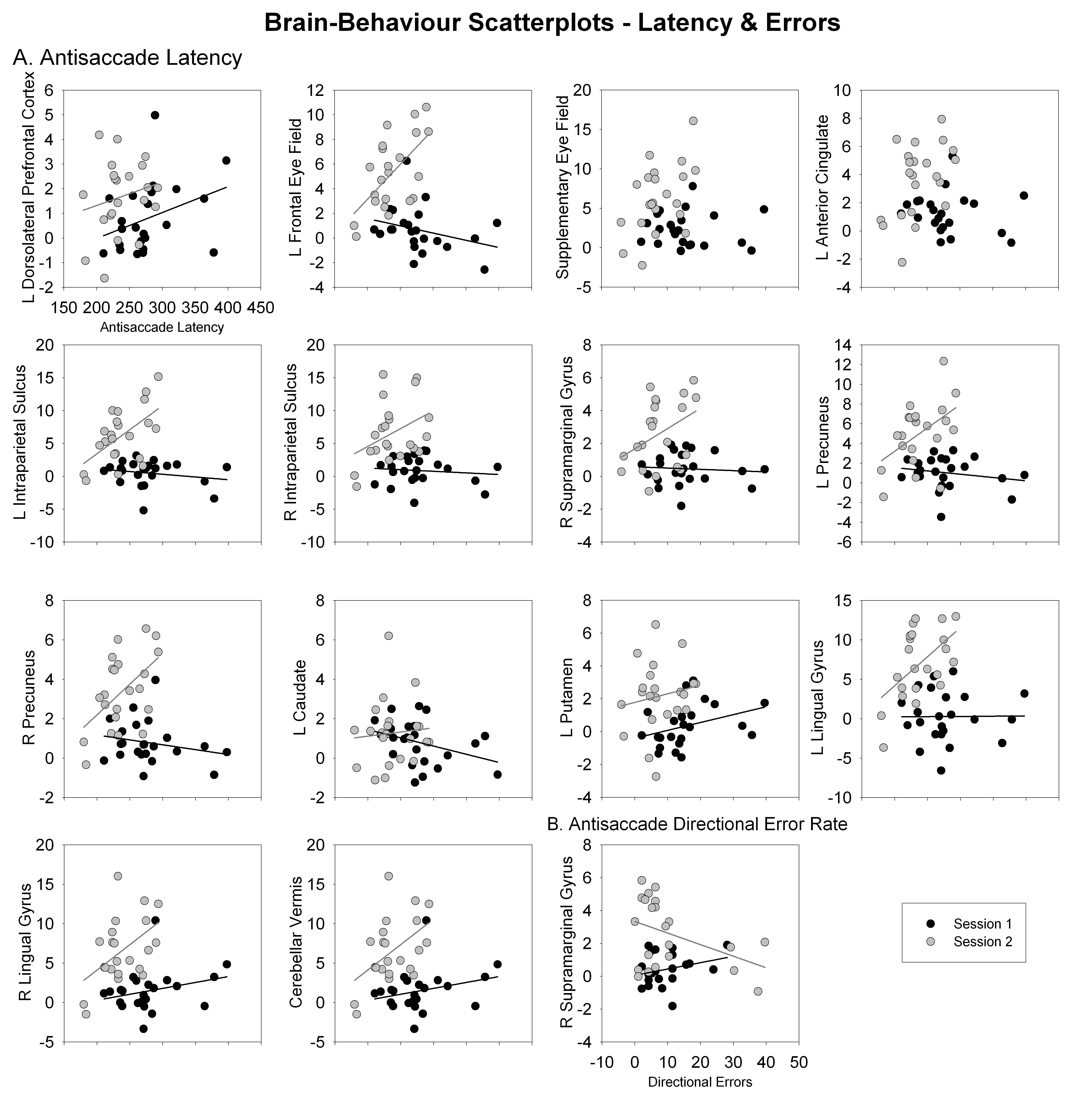


**Fig.** **S2:** (A) Scatterplots of fMRI activity (parameter estimates) and antisaccade latency (milliseconds) for sessions 1 and 2. (B) Scatterplots of fMRI activity (parameter estimates) and antisaccade directional error count for sessions 1 and 2.


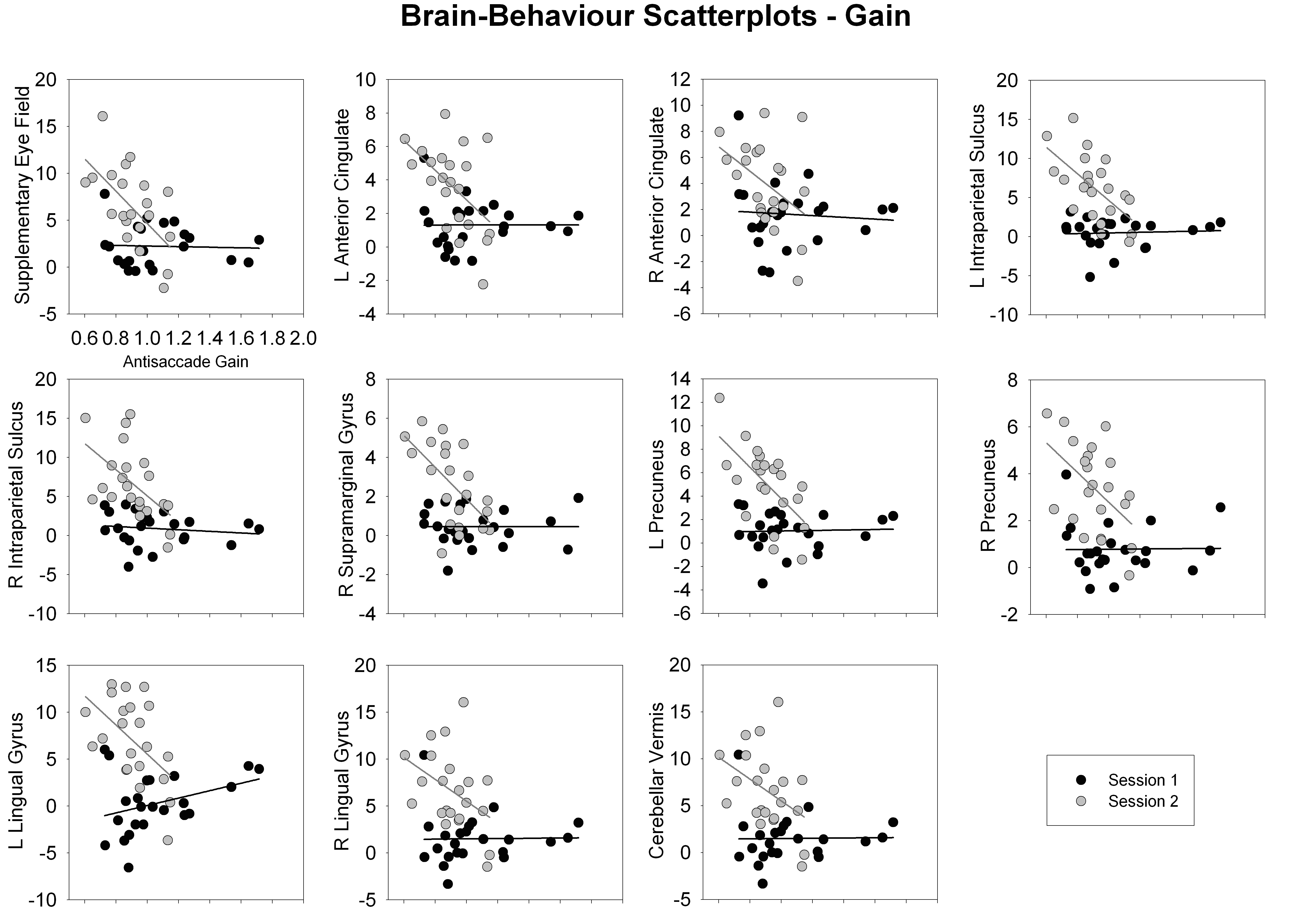


**Fig. S3:** Scatterplots of fMRI activity (parameter estimates) and antisaccade gain for sessions 1 and 2.
